# Review of the genus *Canalirogas* van Achterberg & Chen (Hymenoptera, Braconidae, Rogadinae) from Vietnam, with description of ten new species

**DOI:** 10.3897/zookeys.506.9247

**Published:** 2015-05-28

**Authors:** Khuat Dang Long, Cornelis van Achterberg

**Affiliations:** 1 Institute of Ecology & Biological Resources, Vietnam Academy of Science & Technology, 18 Hoang Quoc Viet Road, Cau Giay, Ha Noi, Vietnam; 2 Department of Terrestrial Zoology, Naturalis Biodiversity Center, Postbus 9517, 2300 RA Leiden, The Netherlands

**Keywords:** Braconidae, Rogadinae, *Canalirogas*, new species, new synonym, key, Vietnam

## Abstract

The Vietnamese species of the genus *Canalirogas* van Achterberg & Chen, 1996 (Hymenoptera: Braconidae: Rogadinae) are revised. Ten species are new to science, viz., *Canalirogas affinis* **sp. n.**, *Canalirogas cucphuongensis* **sp. n.**, *Canalirogas curvinervis* **sp. n.**, *Canalirogas eurycerus* **sp. n.**, *Canalirogas hoabinhicus* **sp. n.**, *Canalirogas intermedius* **sp. n.**, *Canalirogas parallelus* **sp. n.**, *Canalirogas robberti* **sp. n.**, *Canalirogas vittatus* **sp. n.** and *Canalirogas vuquangensis* **sp. n.** One species is new for the Vietnamese fauna: *Canalirogas spilonotus* (Cameron, 1905) and *Canalirogas balgooyi* van Achterberg & Chen, 1996, is synonymized with it (**syn. n.**); a lectotype is designated for *Troporhogas spilonotus*. A key to the Vietnamese species of the genus is also provided.

## Introduction

Little is known about most subfamilies of Braconidae from Vietnam, and the subfamily Rogadinae is no exception. For 15 years specialists of the Institute of Ecology & Biological Resources (IEBR) at Vietnam Academy of Science & Technology (VAST) and RMNH have been collecting Braconidae from all over Vietnam to get a first understanding of the Vietnamese fauna of Braconidae, partly in collaboration with Dr S.A. Belokobylskij (St. Petersburg, Russia). In this paper, the newly discovered species of the Indo-Australian genus *Canalirogas* van Achterberg & Chen, 1996 (Rogadinae) from Vietnam are described. It is a rather small genus comprising eleven known species (Yu et al. 2012), with *Troporhogas spilonotus* Cameron, 1905, from Sri Lanka to be added (Quicke and Shaw 2005). The Vietnamese species were mainly collected using sweep nets and Malaise traps. Specimens of *Canalirogas* mainly occur in more or less open habitats, viz., secondary forest and gardens, as indicated by the pale colour pattern of the body. As far as known, all species of Rogadinae are endoparasitoids of lepidopteran larvae and the larvae are mummified. The checklist and distribution of twenty *Canalirogas* species from the Oriental and Australian regions are given and a key to all known Vietnamese species is provided.

## Material and methods

Most of the examined specimens (including the types) are deposited in the collections of IEBR and VNMN (Ha Noi, Vietnam) and RMNH (Leiden, The Netherlands). The lectotype of *Canalirogas spilonotus* (Cameron) is housed in BMNH (London, UK). The following abbreviations are used: Od = diameter of posterior ocellus; OOL = oculacellar line; POL = postocellar line; MT: Malaise trap; ‘Rog. + number’: code number indexing for specimens of the Rogadinae in the collection; N: North; S: South, NC: North Central, NE: Northeast, NW: Northwest; NP: National Park; IEBR = Institute of Ecology & Biological Resources (Ha Noi, Vietnam), BMNH = Natural History Museum (London, UK), RMNH = Naturalis Biodiversity Center (Leiden, The Netherlands) and VNMN = Vietnam National Museum of Nature (Ha Noi, Vietnam).

For identification of the subfamily, see van Achterberg (1990, 1993, 1997); for the subdivision of the subfamily, see van Achterberg (1991). For separating *Canalirogas* from both similar genera *Macrostomion* Szépligeti and *Colastomion* Baker and for a key to the genera see Chen and He (1997). For the terminology used in this paper, see van Achterberg (1988). Drawings were made under an Olympus SZ40 binocular microscope by the first author. Photographs with scale lines were made with a Canon® G10 camera attached to a Zeiss® 426126 binocular microscope or with a Canon® G15 camera attached to an Olympus® SZ61 binocular microscope by the first author. Those without scale lines were taken with an Olympus SZX12 motorized stereomicroscope with AnalySIS Extended Focal Imaging Software by the second author. Measurements were taken as indicated by van Achterberg (1988).

## Systematics

### Canalirogas

Taxon classificationAnimaliaHymenopteraBraconidae

van Achterberg & Chen, 1996

Figs 1–2 , 3–11 , 12–16 , 17–21 , 22–26 , 27–31 , 32–37 , 38–43 , 44–48 , 49–56 , 57–64 , 65–70 , 71–78

Canalirogas van Achterberg & Chen, 1996: 63–64. Type-species (by original designation): *Canalirogas balgooyi* van Achterberg & Chen, 1996 (examined; = *Canalirogas spilonotus* (Cameron, 1905), **syn. n.**).

#### Diagnosis.

*Canalirogas* can be separated from related genera by the combination of (1) hypopygium of female distinctly convex ventrally and strongly enlarged (Figs 15, 21, 30, 35, 41, 47, 56, 64, 69, 78); (2) ovipositor distinctly curved downwards (Figs 30, 41, 64, 78); (3) ovipositor sheath widened (Figs 15, 25, 30, 47, 56, 64, 78); (4) second metasomal tergite without distinct medio-basal area (Figs 3–4, 6–11, 18, 37, 54, 62, 76); (5) anterior half of fourth-fifth tergites usually (partly) obliquely striate; (6) tarsal claws simple. The vertex is smooth, the prepectal carina complete, the tarsal claws simple, the hind tibia with apical comb on inner side and the dorsope is present.

**Figures 1–2.**
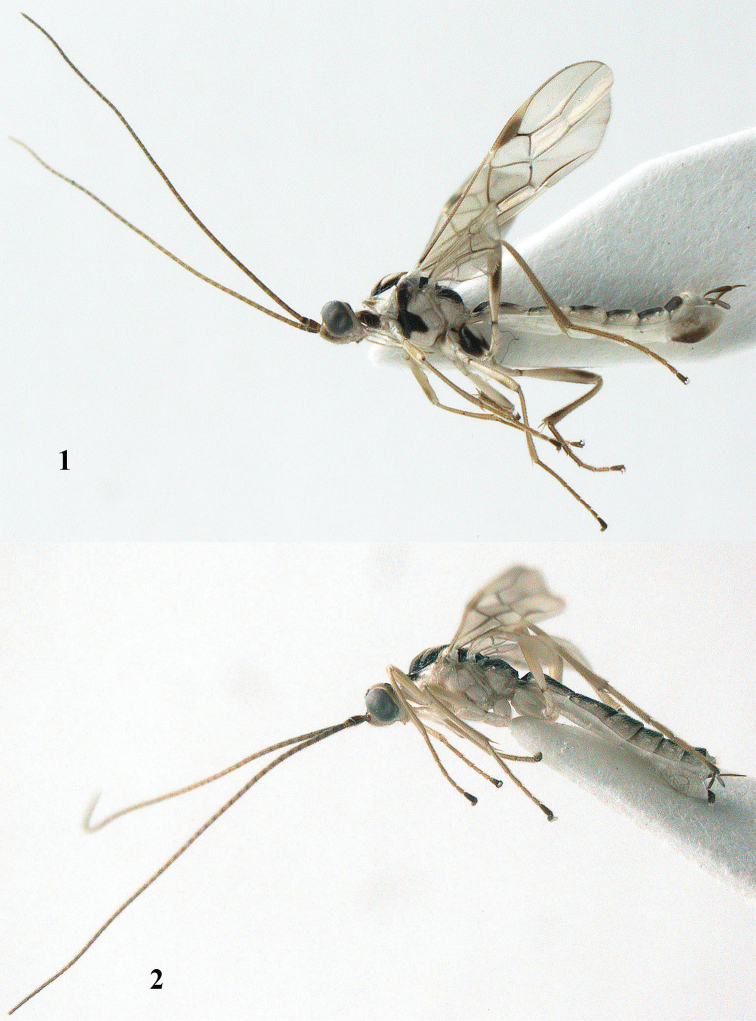
*Canalirogas vuquangensis* sp. n. (**1** holotype, female) and *Canalirogas curvinervis* sp. n. (**2** paratype, female), habitus lateral.

**Figures 3–11.**
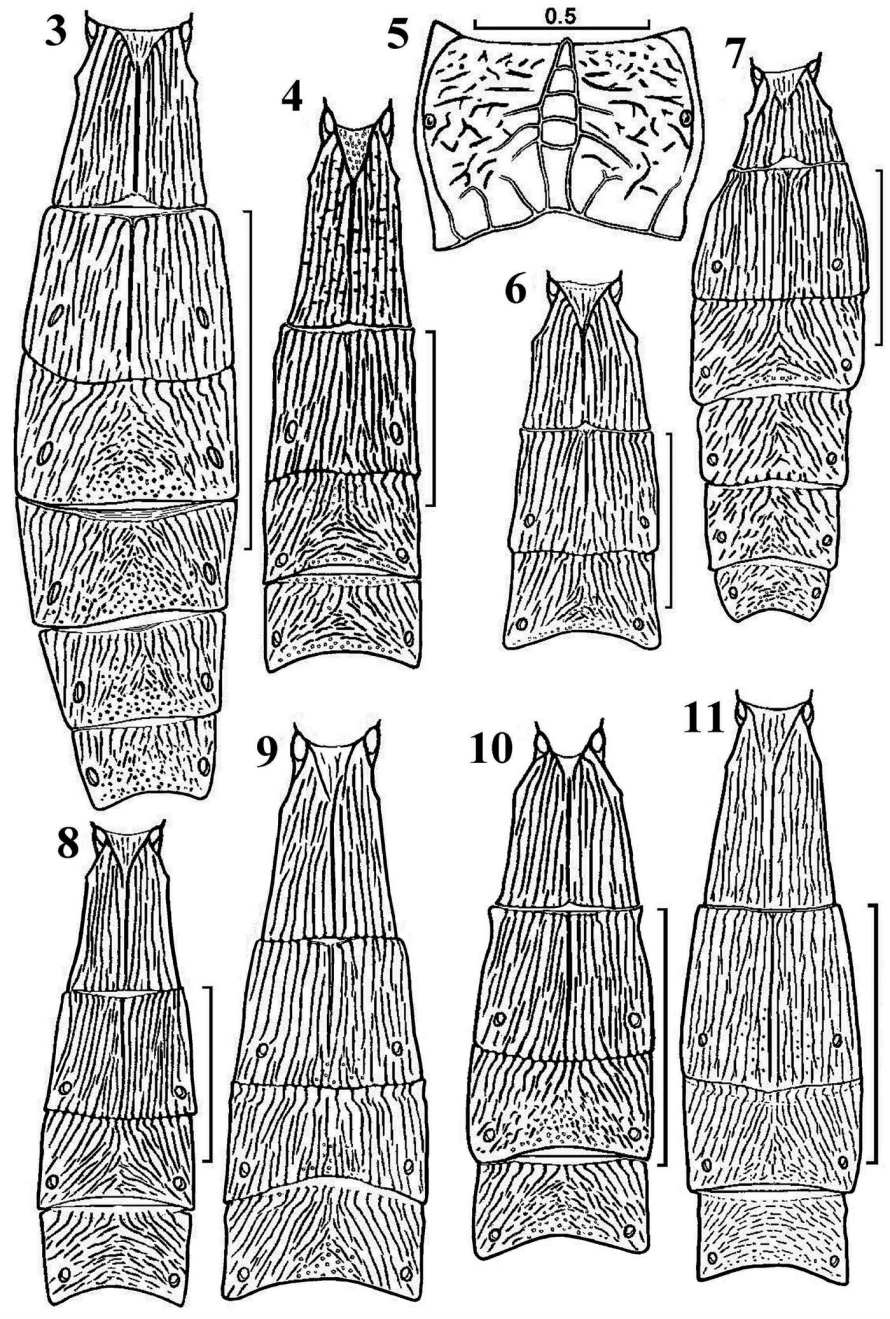
*Canalirogas* spp.: basal metasomal segments dorsal, but 5 propodeum dorsal. **3** *Canalirogas curvinervis* sp. n. **4, 5** *Canalirogas parallelus* sp. n. **6** *Canalirogas hoabinhicus* sp. n. **7** *Canalirogas vittatus* sp. n. **8** *Canalirogas intermedius* sp. n. **9** *Canalirogas affinis* sp. n. **10** *Canalirogas eurycerus* **11** *Canalirogas cucphuongensis* sp. n.

**Figures 12–16.**
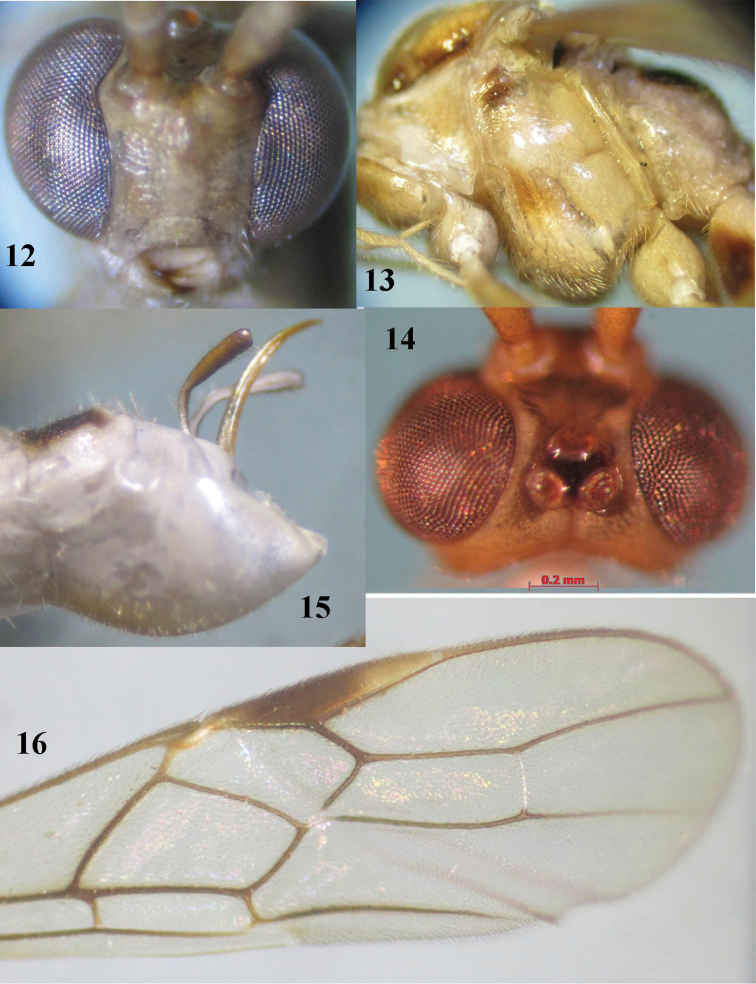
*Canalirogas affinis* sp. n., female, holotype. **12** head anterior **13** mesosoma lateral **14** head dorsal **15** hypopygium lateral **16** apical part of fore wing.

**Figures 17–21.**
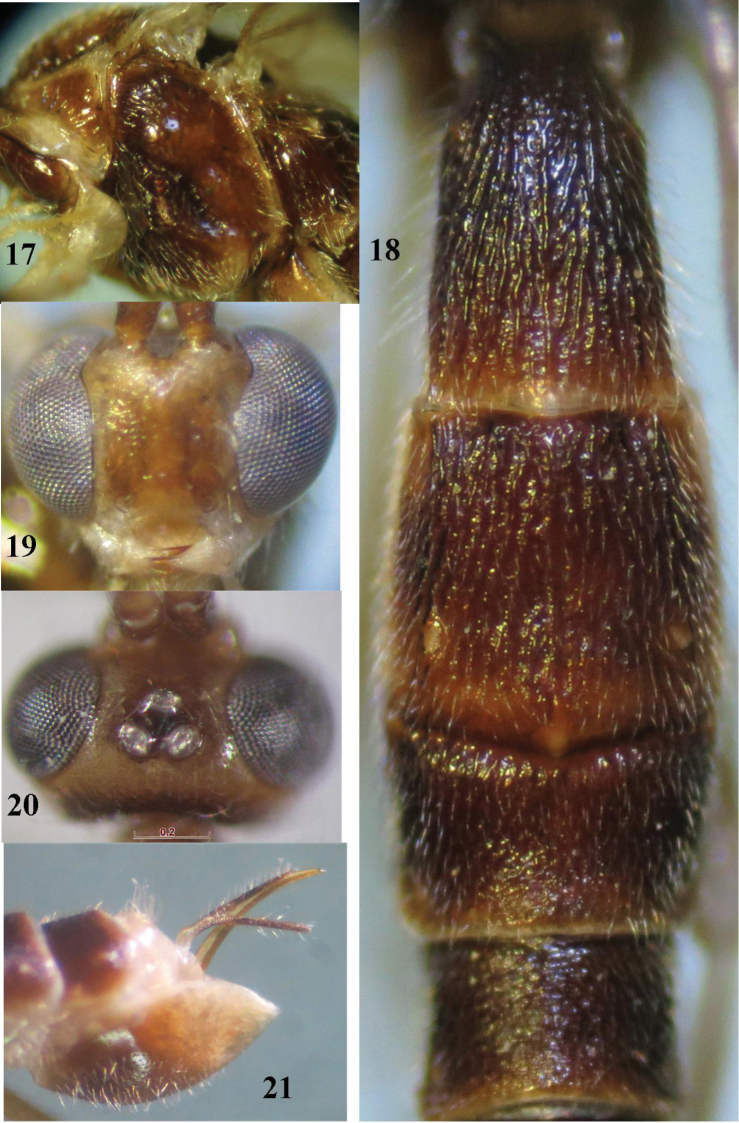
*Canalirogas cucphuongensis* sp. n., female, holotype. **17** mesosoma lateral **18** first-fourth tergites dorsal **19** head anterior **20** head dorsal **21** hypopygium lateral.

**Figures 22–26.**
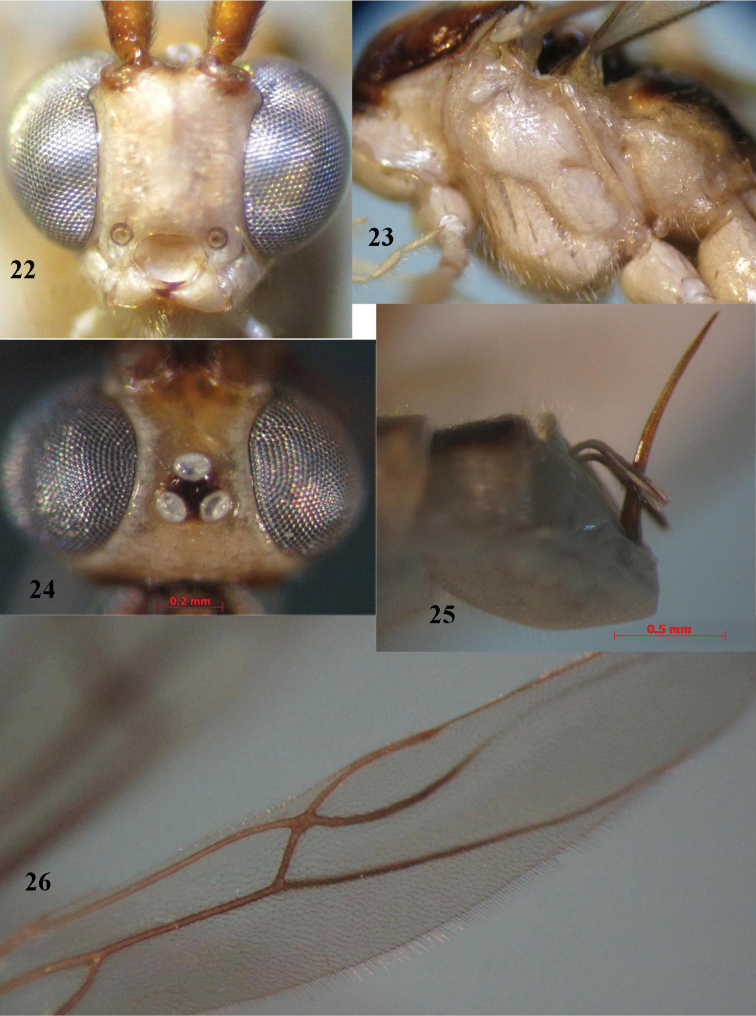
*Canalirogas curvinervis* sp. n., female, holotype. **22** head anterior **23** mesosoma lateral **24** head dorsal **25** hypopygium lateral **26** hind wing.

**Figures 27–31.**
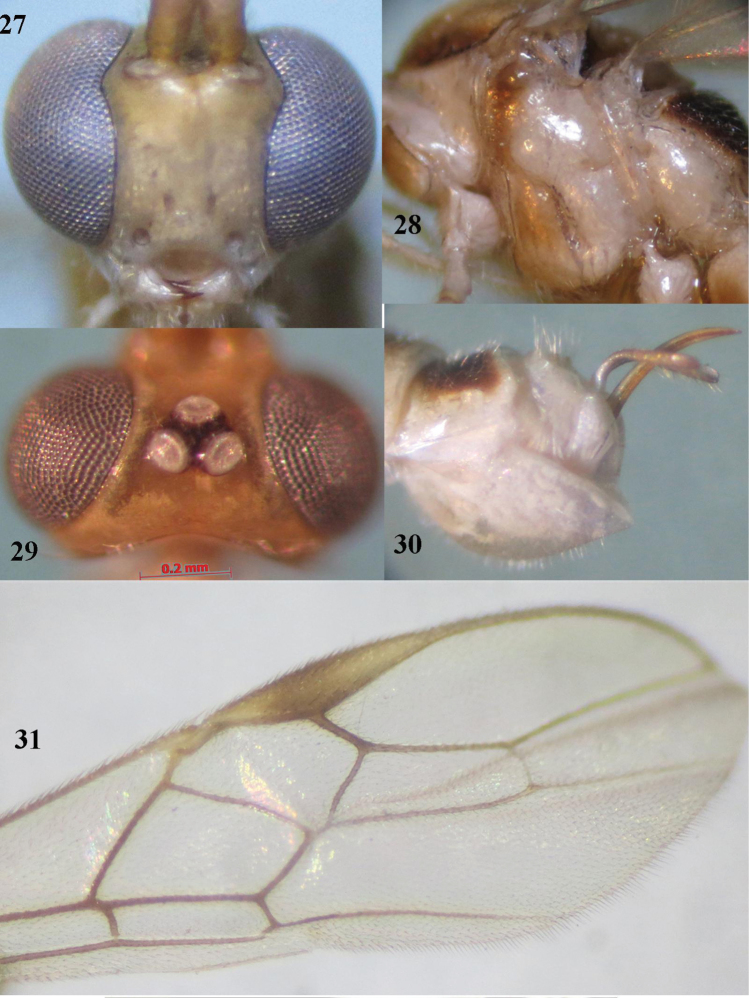
*Canalirogas eurycerus* sp. n., female, holotype. **27** head anterior **28** mesosoma lateral **29** head dorsal **30** hypopygium lateral **31** apical part of fore wing.

**Figures 32–37.**
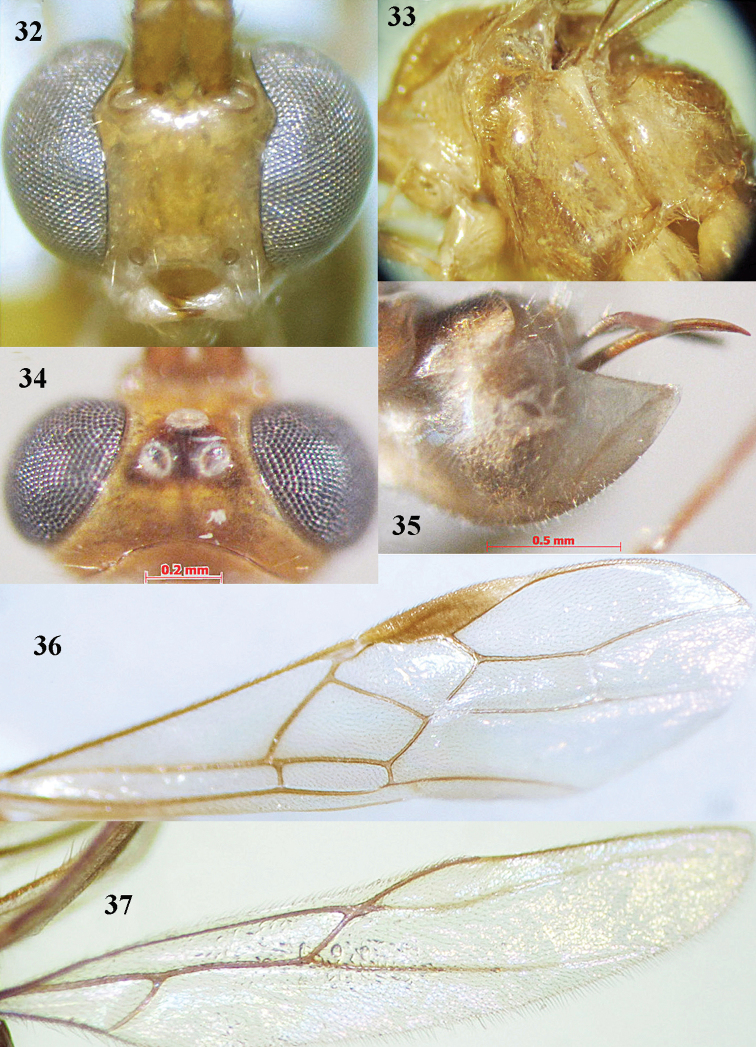
*Canalirogas hoabinhicus* sp. n., female, holotype. **32** head anterior **33** mesosoma lateral **34** head dorsal **35** hypopygium lateral **36** fore wing **37** hind wing.

**Figures 38–43.**
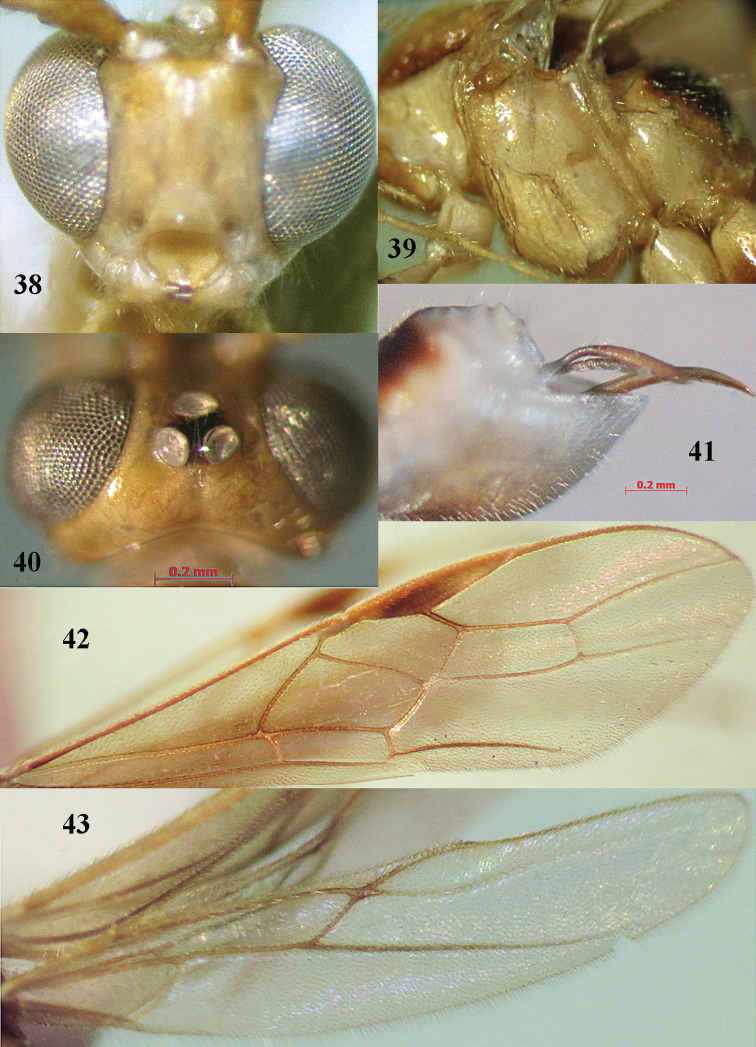
*Canalirogas intermedius* sp. n., female, holotype. **38** head anterior **39** mesosoma lateral **40** head dorsal **41** hypopygium lateral **42** fore wing **43** hind wing.

**Figures 44–48.**
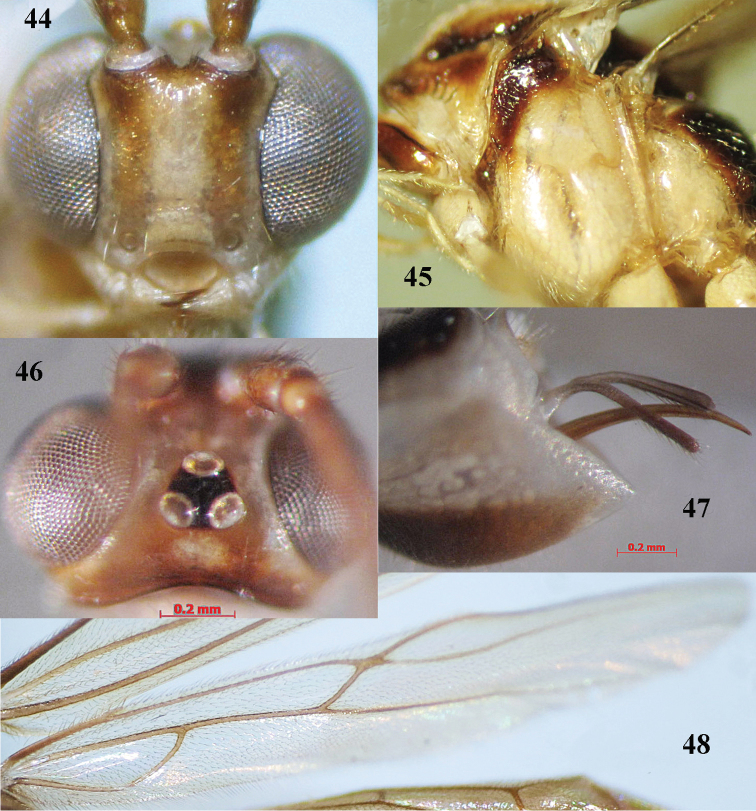
*Canalirogas parallelus* sp. n., female, holotype. **44** head anterior **45** mesosoma lateral **46** head dorsal **47** hypopygium lateral **48** hind wing.

**Figures 49–56.**
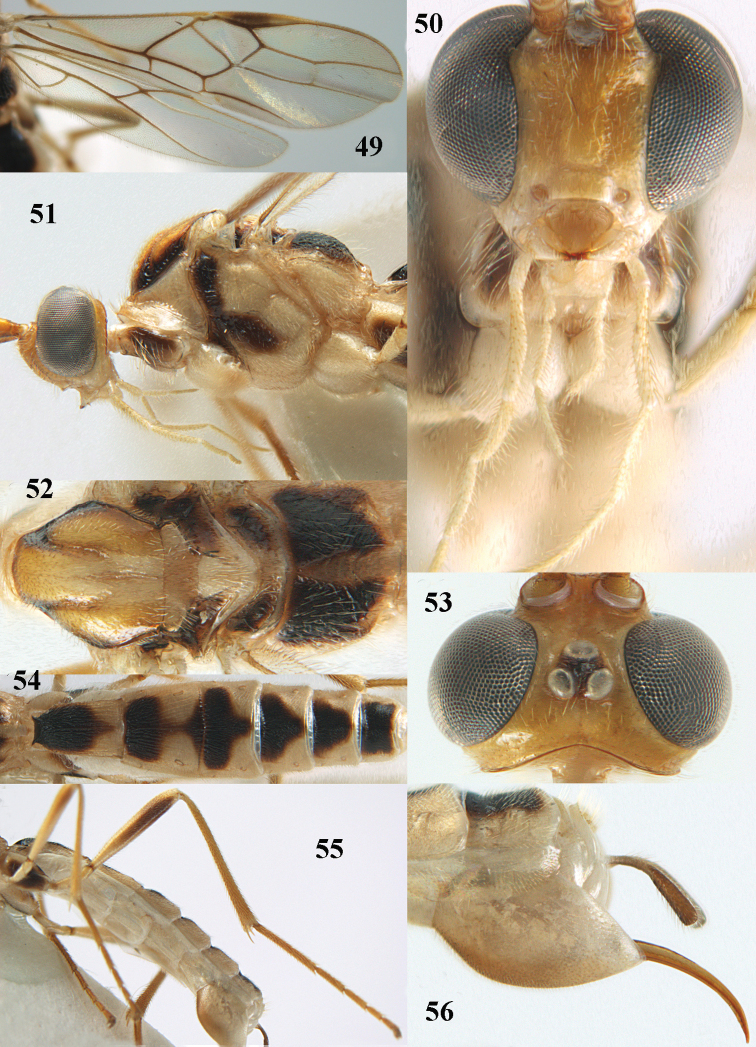
*Canalirogas robberti* sp. n., female, holotype. **49** wings **50** head anterior **51** head and mesosoma lateral **52** mesosoma dorsal **53** head dorsal **54** first-sixth tergites dorsal **55** hind leg lateral **56** hypopygium lateral.

**Figures 57–64.**
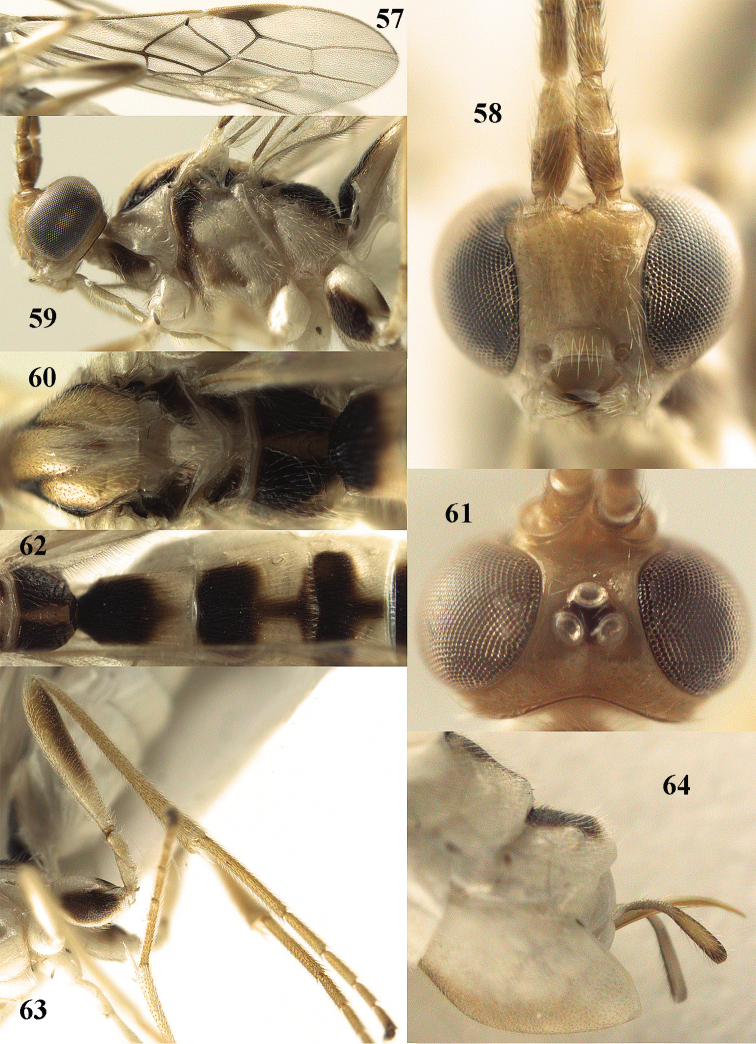
*Canalirogas spilonotus* (Cameron), female, Vietnam. **57** fore wing **58** head anterior **59** head and mesosoma lateral **60** mesosoma dorsal **61** head dorsal **62** propodeum and first-third tergites dorsal **63** hind leg lateral **64** hypopygium lateral.

**Figures 65–70.**
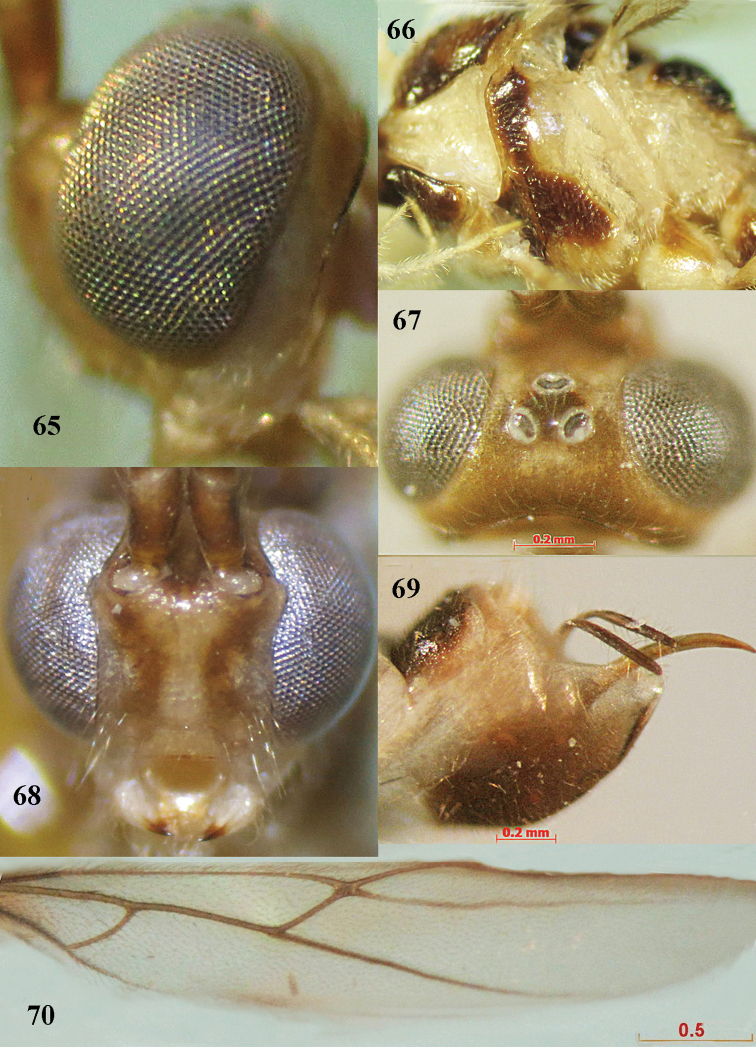
*Canalirogas vittatus* sp. n., female, holotype. **65** head lateral **66** mesosoma lateral **67** head dorsal **68** head anterior **69** hypopygium lateral **70** hind wing.

**Figures 71–78.**
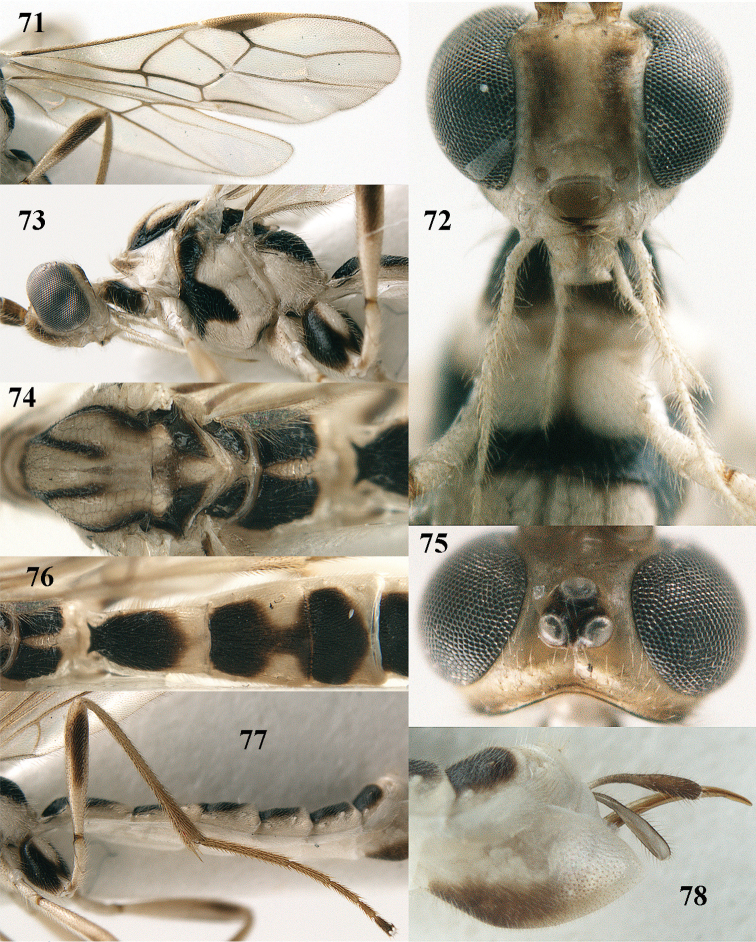
*Canalirogas vuquangensis* sp. n., female, holotype. **71** wings **72** head anterior **73** head and mesosoma lateral **74** mesosoma dorsal **75** head dorsal **76** propodeum and first-third tergites dorsal **77** hind leg lateral **78** hypopygium lateral.

#### Biology.

Parasitoids of Lymantriidae on clove trees (Quicke and Shaw 2005).

#### Checklist and distribution

*Canalirogas acutus* van Achterberg, 1996, from Indonesia, Malaysia

*Canalirogas agilis* van Achterberg, 1996, from Indonesia

*Canalirogas affinis* sp. n., from Vietnam

*Canalirogas cucphuongensis* sp. n., from Vietnam

*Canalirogas curvinervis* sp. n., from Vietnam

*Canalirogas eurycerus* sp. n., from Vietnam

*Canalirogas fuscipalpis* van Achterberg, 1996, from Indonesia

*Canalirogas heijningeni* van Achterberg, 1996, from Indonesia

*Canalirogas hoabinhicus* sp. n., from Vietnam

*Canalirogas infuscatus* van Achterberg, 1996, from Malaysia

*Canalirogas intermedius* sp. n., from Vietnam

*Canalirogas kahonoi* van Achterberg, 1996, from Indonesia, Malaysia

*Canalirogas maculatus* van Achterberg, 1996, from Indonesia

*Canalirogas nigratus* van Achterberg, 1996, from Indonesia

*Canalirogas parallelus* sp. n., from Vietnam

*Canalirogas robberti* sp. n., from Vietnam

*Canalirogas spilonotus* (Cameron, 1905), from Sri Lanka and including *Canalirogas balgooyi* van Achterberg & Chen, 1996, from Burma, China, India, Indonesia, Malaysia, Nepal, Vietnam. Syn. n.

*Canalirogas tuberculatus* van Achterberg, 1996, from Indonesia

*Canalirogas vittatus* sp. n., from Vietnam

*Canalirogas vuquangensis* sp. n. from Vietnam

*Canalirogas yvonnae* van Achterberg, 1996, from Indonesia, Malaysia

#### Key to Vietnamese species of the genus *Canalirogas* van Achterberg & Chen

**Table:** 

1	Second metasomal tergite about twice as long as third tergite medially (Figs 11, 18); mesosternum behind prepectal (= epicnemial) carina; mesopleuron medially dark brown or reddish brown (Fig. 17)	***Canalirogas cucphuongensis* sp. n.**
–	Second tergite 1.5–1.8 times longer than third tergite medially (Figs 3, 4, 6–10, 62, 76); mesosternum (except more or less anteriorly) and mesopleuron medially yellowish-brown, pale yellow or ivory (Figs 2, 13, 23, 28, 33, 39, 45, 51, 59, 66)	**2**
2	Hind tarsus mainly dark brown; first metasomal tergite hardly longer than wide apically and distinctly widened apically (Fig. 7); hypopygium of female largely dark brown ventrally (Fig. 69)	***Canalirogas vittatus* sp. n.**
–	Hind tarsus mainly yellowish-brown (usually except dark brown or brown telotarsus); first tergite 1.2–2.1 times longer than wide apically and usually slightly widened or parallel-sided (Figs 3, 4, 6, 8–10, 62, 76); hypopygium of female at least partly pale yellowish ventrally (Figs 30, 41, 56, 64), but brownish in *Canalirogas parallelus* and *Canalirogas vuquangensis* (Figs 47, 78)	**3**
3	Basal third of vein SR of hind wing sclerotised and distinctly curved (Fig. 26); hypopygium of female less convex baso-ventrally (Fig. 25); clypeus flattened; ovipositor nearly straight (Fig. 25); second metasomal tergite mainly blackish or dark brown	***Canalirogas curvinervis* sp. n.**
–	Basal third of vein SR of hind wing only pigmented and slightly curved (Figs 37, 43, 48, 49); hypopygium of female distinctly convex baso-ventrally (Figs 30, 35, 56); clypeus convex or concave (Figs 12, 50, 58, 72); ovipositor distinctly curved (Figs 35, 56, 69, 78); second metasomal tergite partly yellowish-brown or pale yellowish	**4**
4	First metasomal tergite gradually widened subapically, first tergite 1.2–1.3 times longer than its apical width (Figs 6, 10); outer side of hind femur pale yellowish, at most partly infuscate; hypopygium yellow or ivory ventrally (Fig. 30); ovipositor rather slender (Figs 30, 35)	**5**
–	First tergite subparallel-sided, first tergite 1.4–1.9 times as long as apical width (Figs 4, 8, 9, 54), if apically somewhat widened, outer side of hind femur usually partly distinctly infuscate, dark yellowish brown or dark brown (Figs 55, 63, 77); ovipositor less slender (Figs 56, 69, 78)	**6**
5	Ocelli smaller (Fig. 34), diameter of posterior ocellus of female 1.2–1.3 times POL; second segment of maxillary palp widened medially; hind femur about 6 times as long as wide, its outer side without infuscation; vertex partly (Fig. 34) and mesoscutum pale brown; propodeum brown laterally (Fig. 33); vein r of fore wing slender (Fig. 36)	***Canalirogas hoabinhicus* sp. n.**
–	Ocelli larger (Fig. 29), diameter of posterior ocellus of female about 2.3 times POL; second segment of maxillary palp normal medially; hind femur about 5 times as long as wide, its outer side partly infuscate; vertex (Fig. 29) and mesoscutum pale yellowish; propodeum dark brown laterally (Fig. 28); vein r of fore wing widened (Fig. 31)	***Canalirogas eurycerus* sp. n.**
6	Apex of third tergite without medio-apical divergent striation (Fig. 9); mesopleuron behind prepectal carina mainly brownish-yellow, without dark brown patch (Fig. 13); hypopygium mainly ivory or somewhat pale yellow	***Canalirogas affinis* sp. n.**
–	Apex of third tergite with more or less medio-apical divergent striation (Figs 4, 6–8, 10, 62); mesopleuron behind prepectal carina brownish yellow or dark brown (Figs 28, 51, 59, 66, 73), if not, hypopygium dark brown basally (Fig. 41) or first-second metasomal tergites mainly dark brown (Figs 54, 76)	**7**
7	Propodeum entirely dark brown or black, without pale area medially; outer side of hind femur and of hind coxa yellow; side of pronotum dark brown dorsally (Fig. 45)	***Canalirogas parallelus* sp. n.**
–	Propodeum with yellowish longitudinal area medially (Figs 52, 74) and remainder brown or dark brown; outer side of hind femur more or less dark brown subapically; colour of hind coxa variable; side of pronotum ivory or pale yellow dorsally (Figs 51, 59, 73)	**8**
8	Clypeus shallowly concave medially; ventral rim differentiated and slightly protruding (Fig. 51); [first metasomal tergite 1.7–1.9 times as long as wide apically and parallel-sided apically (Fig. 54)]	***Canalirogas robberti* sp. n.**
–	Clypeus flat medially or slightly convex; ventral rim absent (Figs 59, 73)	**9**
9	Third and fourth metasomal tergites with sub-transverse elements posteriorly (Fig. 8); apical width of first tergite 1.7–1.8 times its minimum subbasal width (Fig. 8); diameter of posterior ocellus about 1.4 times OOL; occipital carina evenly concave (Fig. 40)	***Canalirogas intermedius* sp. n.**
–	Third and fourth tergites without sub-transverse elements posteriorly and only obliquely striate (Figs 62, 76); apical width of first tergite 1.5–1.6 times its minimum subbasal width (Figs 62, 76); diameter of posterior ocellus usually 2–3 times OOL (Figs 61, 75); occipital carina deeply concave (Fig. 75)	**10**
10	Area in front of prepectal carina pale yellow (Fig. 59); face yellow sublaterally (Fig. 58); ovipositor sheath mainly yellowish brown and only apically darkened (Fig. 64); temple narrow, eye in lateral view about 6 times as wide as temple (Fig. 59)	***Canalirogas spilonotus* (Cameron, 1905)**
–	Area in front of prepectal carina dark brown (Fig. 73); head brown sublaterally (Fig. 72); ovipositor sheath mainly dark brown (Fig. 78); temple somewhat wider, eye in lateral view about 4.4 times as wide as temple (Fig. 73)	***Canalirogas vuquangensis* sp. n.**

### Descriptions

#### Canalirogas affinis sp. n.

Taxon classificationAnimaliaHymenopteraBraconidae

http://zoobank.org/DC0F9921-0428-47A3-9DCA-75EF6AF7E505

Figs 9 , 12–16

##### Material.

Holotype, female (VNMN), ‘Rog.639’, “[NE Vietnam:] Phu Tho, Tan Son, Kiet Son, orchard, 21°10'N, 104°57E', 110 m, MT, 11-15.v.2009, KD Long, NH Thao”. Paratypes (13 females; VNMN, RMNH): 1 female, ‘Rog.632’, id. but 6–10.vii.2009; 1 female, ‘Rog.636’, id. but 6–10.v.2009; 1 female, ‘Rog.637’, id. but 16–20.vi.2009; 1 female, ‘Rog.638’, id. but 20–26.vii.2009; 1 female, ‘Rog.640’, id. but 6–10.vi.2009; 1 female, ‘Rog.641’, id. but 26–30.vii.2009; 1 female, but ‘Rog.643’, id. 26–31.vii.2009; 1 female, ‘Rog.647’, id. but 1–5.vii.2009; 1 female, ‘Rog.648’, id. but 15–31.viii.2009; 1 female, ‘Rog.772’, “[NE Vietnam:] Phu Tho, Tan Son, Xuan Dai, 21°07'N, 105°00'E, 120 m, MT 11–15.iv.2009, KD Long; 1 female, ‘Rog.471’, “[NE Vietnam:] Vinh Phuc, Me Linh, Ngoc Thanh, forest, 21°23'N, 105°43'E, 280 m, MT, 1–13.viii.2000, KD Long”; 1 female, ‘Rog.663’, id. but MT 23.v.-7.vi.2001, KD Long; 1 female, ‘Rog.644’, id. but MT, 4–15.v.2001, KD Long.

##### Description.

Holotype, female, body length 7.7 mm, fore wing length 5.7 mm.

*Head.* Antenna incomplete, with 45 segments remaining; middle segments 3.2 times longer than wide (8:2.5); third antennal segment 1.25 times fourth (10:8); width of face 0.8 times length of face and clypeus combined (18:22); malar space 0.7 times as long as mandible width (5:7); mandible width 0.7 times as long as hypoclypeal depression (7:10); malar suture present; distance between tentorial pits 3.3 times distance between pits and eyes (10:3) (Fig. 12); in dorsal view height of eye 5.0 times as long as temple (20:4); in lateral view width of eye 4.5 times longer than temple (18:4); ocelli large, POL:Od:OOL = 1:2:1; distance between front and hind ocelli as long as OOL (Fig. 14); face sparsely rugose; frons, vertex and temple smooth.

*Mesosoma.* Length of mesosoma 1.4 times longer than high (81:58); pronotal side smooth dorsally, crenulate medially, finely granulate ventrally; mesoscutum smooth; notauli rather shallow, flat posteriorly and smooth; precoxal sulcus short, nearly smooth; mesopleuron and metapleuron smooth (Fig. 13); scutellar sulcus 0.8 times as long as scutellum (7:9); scutellum smooth; propodeum rugose-punctate laterally, median areola with median transverse carinae.

*Wings.* Fore wing: pterostigma 5.2 times longer than wide (52:10); r:2-SR:3-SR:SR1=12:9:32:38; vein r arising little before middle of pterostigma; vein cu-a short and robust (Fig. 16), 1-CU1:cu-a:2-CU1:3-CU1=3:7:31:6; posterior length of second submarginal cell 2.7 times its apical width (40:15). Hind wing: vein M+CU:1-M: 1r-m=32:30:11.

*Legs.* Hind coxa shining with sparse fine punctures; length of hind femur:tibia:basitarsus: tarsus = 32:43:22:50; length of hind femur, tibia and basitarsus 5.3, 9.5 and 11.0 times as long as their width respectively; inner hind tibial spur 0.25 times as long as basitarsus (11:44).

*Metasoma.* First tergite 1.45 times as long as apical width (43:32) (Fig. 9); first-third tergites with parallel striation; fourth tergite with divergent striation; medial length of second tergite 1.5 times than third (33:22); second suture crenulate; ovipositor sheath truncate apically, 0.5 times as long as hind basitarsus (22:44); ovipositor stout (Fig. 15).

*Colour.* Pale yellow; antenna yellow; palpi whitish yellow; stemmaticum black; pronotum, mesopleuron, metapleuron pale yellow; hind coxa blackish dorsally and ventrally, yellow basally and ventrally; propodeum laterally, metasomal tergites 1-5 basally and tergite 6 entirely, black; wing subhyaline with brownish yellow veins, setae pale yellow; pterostigma dark brown medially, yellow basally and apically (Fig. 16).

##### Male.

Unknown.

##### Variation.

Paratypes, antenna with 44–52 segments; first tergite 1.35–1.45 times as long as apical width; medial length of metasomal second tergite 1.5–1.6 times as long as third tergite medially; body length 5.6–8.0 mm; fore wing length 4.6–5.9 mm.

##### Etymology.

Named ‘affinis’ (Latin for ‘related to’), because this species is close to *Canalirogas spilonotus* (Cameron).

#### Canalirogas cucphuongensis sp. n.

Taxon classificationAnimaliaHymenopteraBraconidae

http://zoobank.org/C57127C0-6DF6-44CB-8167-54125B7E4EB0

Figs 11 , 17–21

##### Material.

Holotype, female (VNMN), ‘Rog.202’, “[NW Vietnam:] Hoa Binh, Yen Thuy, secondary forest close to Cuc Phuong NP, 20°28'N, 105°34'E, 315 m, M[alaise] T[rap], 10–20.vi.2002, KD Long”.

##### Description.

Holotype, female, body length 5.7 mm, fore wing length 4.0 mm, antenna 6.7 mm, ovipositor sheath 0.4 mm.

*Head.* Antenna with 47 segments, 1.2 times longer than body; third antennal segment 1.14 times fourth one (8:7); middle segments 3.0 times as long as wide (6:2), penultimate antennal segment 0.6 times apical segment; apical segment with spine; width of face 0.9 times length of face and clypeus combined (15:16); malar space 0.8 times as long as mandible width (4:5), mandible width 0.6 times as long as hypoclypeal depression (5:9); malar suture present; distance between tentorial pits 2.3 times distance between pits and eyes (7:3; Fig. 19); in dorsal view height of eye 3.5 times as long as temple (14:4); in lateral view width of eye 2.4 times as long as temple (12:5); ocelli in high triangle, POL:Od:OOL = 4:6:5, distance between front and hind ocelli as long as OOL (Fig. 20); face rugose laterally, smooth medially; frons, vertex and temple smooth.

*Mesosoma.* Length of mesosoma 1.4 times as long as high (57:41); pronotal trough smooth dorsally, crenulate medially, finely granulate ventrally; precoxal sulcus short, narrow and crenulate (Fig. 17); mesopleuron and metapleuron shiny and smooth; notauli deep and crenulate, united posteriorly in a deep groove; mesoscutum with sparse fine punctures; scutellum almost smooth; scutellar sulcus 0.9 times as long as scutellum (10:11); propodeum punctate basally, rugose apically, medial areola crenulate.

*Wings.* Fore wing: pterostigma 4.4 times as long as wide (35:8); r:2-SR:3-SR:SR1 = 7:9:21:36; vein r before middle of pterostigma; vein cu-a short and vertical, vein 1-CU1 quadrate; posterior length of second submarginal cell 3.3 times its apical width (30:9). Hind wing: vein M+CU:1-M:1r-m = 22:18:10.

*Legs.* Hind coxa smooth; length of hind femur:tibia:basitarsus:tarsus = 51:63:34:85; length of hind femur, tibia and basitarsus 5.7, 10.5 and 11.3 times their width, respectively; inner hind tibial spur 0.2 times as long as basitarsus (7:34).

*Metasoma.* First tergite 1.5 times longer than apical width (32:21) (Figs 11, 18); second suture more or less crenulate; medial length of metasomal second tergite 2.2 times as long as third tergite medially (29:13); second-third tergites with comparatively less divergent striation (Fig. 18); fourth-fifth tergites with curved striation; sixth tergite granulate; ovipositor sheath 0.8 times as long as hind basitarsus (28:34); ovipositor slightly curved (Fig. 21).

*Colour.* Brown; head and antenna yellow; palpi yellow; stemmaticum brown; fore and middle legs yellow; hind coxa, hind femur subapically and hind telotarsus brownish; metasoma ventrally yellow; wings subhyaline, with veins brownish, but parastigma yellow; mesosternum dark brown and mesopleuron dark or reddish brown (Fig. 17).

##### Male.

Unknown.

##### Etymology.

Named after the famous national park near its type locality: Cuc Phuong National Park.

#### Canalirogas curvinervis sp. n.

Taxon classificationAnimaliaHymenopteraBraconidae

http://zoobank.org/B4C391B4-C285-4107-BD3D-BBDB62661254

Figs 2 , 3 , 22–26

##### Material.

Holotype, female (RMNH) “[C Vietnam:] Thua Thien-Hue, Phong Dien N.R., 15 km W. Phong My, 80–210 m, 23.iii.-6.iv.2001, Mal. trap, C. v. Achterberg & R. de Vries, RMNH’01”. Paratypes (5 females): 1 female (IEBR), “[NE Vietnam:] Viet Tri, nr Thanh Son, Thuong Cuu, 20°59'N, 105°8'E, 350–400 m, 11–16.x.1999, Mal. trap, R. de Vries, RMNH’99”; 1 female (RMNH), ‘Rog.662’, “[NE Vietnam:] Vinh Phuc, Tam Dao NP, 200 m, MT, 23.v.-7.vi.2001, KD Long”; 1 female (RMNH), “[NC Vietnam:] Ha Tinh, Vu Quang NP, 94 m, 18°17'43"N, 105°25'49"E, 5.iii.–15.iv.2011, Mal. trap 13, C. v. Achterberg, RMNH’11”; 1 female (VNMN), ‘Rog.690’, “[NC Vietnam:] Ha Tinh, Vu Quang NP, 6.x.2009, KD Long”; 1 female (RMNH), “[S Vietnam:] Dông Nai, Cát Tiên N.P., Bird trail, Mal. trap[s] 9–12, c. 100 m, 1–9.x.2005, C. v. Achterberg & R. de Vries, RMNH’05”.

##### Description.

Holotype, female, body length 7.2 mm, fore wing length 5.3 mm.

*Head.* Antenna with 28 segments remaining; third antennal segment 1.1 times fourth one (10:9); middle segments 2.3 times its width (7:3); width of face 0.9 times length of face and clypeus combined (19:21); malar space as long as mandible width; mandible width 0.6 times as long as hypoclypeal depression (6:10); malar suture present; distance between tentorial pits 5.0 times distance between pits and eyes (10:2; Fig. 22); in dorsal view height of eye 5.8 times as long as temple (23:4); in lateral view width of eye 3.2 times as long as temple (19:6); ocelli in high triangle, POL:Od:OOL = 3:6:4; distance between front and hind ocelli:OOL = 3:4 (Fig. 24); face rugose; frons, vertex and temple smooth.

*Mesosoma.* Length of mesosoma 1.5 times as long as high (90:60); pronotal side smooth dorsally, crenulate anteriorly, finely granulate ventrally; precoxal sulcus long, deep and crenulate (Fig. 23); mesopleuron smooth; mesoscutum smooth; notauli deep and crenulate; propodeum mainly rugose and without distinct medial areola.

*Wings.* Fore wing: pterostigma 4.8 times as long as wide; r:2-SR:3-SR:SR1=10:18:28:50; vein r arising submedially from pterostigma (Fig. 28); vein 1-CU1 quadrate; vein cu-a perpendicular; posterior length of second submarginal cell 3.2 times its width (38:12). Hind wing: vein M+CU as long as vein 1-M and 3.5 times vein 1r-m = 35:10 (Fig. 26).

*Legs.* Hind coxa smooth; length of hind femur:tibia:basitarsus:tarsus = 71:92:13:43; length of hind femur, tibia and basitarsus 5.9, 10.2 and 10.75 times their width, respectively.

*Metasoma.* First-second tergites with median carinae and parallel striae; first tergite 1.2 times as long as its apical width (Fig. 3); medial length of second tergite 1.6 times third (42:27); third-fifth tergites basally with divergent striation, granulate medio-apically; sixth tergite mainly granulate; ovipositor sheath pointed apically (0.5 mm), 0.4 times as long as hind basitarsus (20:46); ovipositor slender, nearly straight, enlarged basally (Fig. 25).

*Colour.* Yellow; antenna yellowish, but darkened basally; palpi pale yellow; stemmaticum black; pronotum dorsally, notauli and mesonotum laterally, side of scutellum and axilla, propodeum medially blackish-brown or black; first-sixth tergites black medially, pale yellow laterally; wings subhyaline.

##### Male.

Unknown

##### Variation.

Antenna with 45(1) and 49(1) segments and 1.4 times as long as body; first tergite 1.1–1.3 times as long as apical width; medial length of metasomal second tergite 1.7–1.8 times as long as third tergite medially; body length 5.2–5.9 mm; fore wing length 4.3–4.5 mm.

##### Etymology.

From ‘curvus’ (Latin for bend), because of the curved vein SR of hind wing.

#### Canalirogas eurycerus sp. n.

Taxon classificationAnimaliaHymenopteraBraconidae

http://zoobank.org/494603D1-0CE9-45EA-9DE1-968E8821E318

Figs 10 , 27–31

##### Material.

Holotype, female (VNMN), ‘Rog.469’, “[C Vietnam:] Thua Thien-Hue, Nam Dong, MT, 2–6.v.2005, NQ Truong”. Paratypes (2 females): 1 female (VNMN), ‘Rog.076’, “[C Vietnam:] Ha Tinh, Huong Son 18°22'N, 105°13'E, 200 m, 15.v.1998, MT, AMNH, K Long”; 1 female (RMNH), ‘Rog.642’, “[NE Vietnam:] Phu Tho, Tan Son, 21°14'N, 104°57'E, 140 m, MT, 16–20.vii.2009, NH Thao”.

##### Description.

Holotype, female, body length 5.4 mm, fore wing length 3.9 mm.

*Head.* Antenna incomplete, with 37 segments remaining; third antennal segment as long as fourth; middle segments 2.8 times its width; width of face 0.8 times length of face and clypeus combined (13:16); malar space 0.8 times as long as mandible width (4:8); mandible width 0.7 times as long as hypoclypeal depression (5:7); malar suture present; distance between tentorial pits 3.5 times distance between pits and eyes (7:2; Fig. 27); in dorsal view height of eye 5.3 times as long as temple (16:3); temple narrow, in lateral view width of eye 4.7 times as long as temple (14:3); ocelli large, POL:Od:OOL=3:7:4 (Fig. 29); distance between front and hind ocelli:OOL = 3:4; face mostly smooth with sparse fine punctures; frons, vertex and temple smooth.

*Mesosoma.* Length of mesosoma 1.2 times as long as high (47:38); pronotal trough mainly smooth, sparsely crenulate anteriorly; precoxal sulcus short, deep and smooth; mesopleuron and metapleuron smooth (Fig. 28); mesoscutum and scutellum smooth; notauli short, crenulate anteriorly, flat and smooth posteriorly; propodeum rugose laterally with medial areola crenulate.

*Wings.* Fore wing: pterostigma 4.4 times as long as wide; r:2-SR:3-SR:SR1 = 8:11:22:31; vein r arising before middle of pterostigma (Fig. 31); 1-CU1:cu-a:2-CU1:3-CU1 = 2:4:21:4; posterior length of second submarginal cell 3.0 times its width (30:10). Hind wing: vein M+CU 1.1 times vein 1-M (24:22) and 3.0 times vein 1r-m (24:8).

*Legs.* Hind coxa smooth; length of hind femur:tibia:basitarsus:tarsus = 44:55:28:68; length of hind femur, tibia and basitarsus 4.9, 9.2 and 14.0 times their width, respectively; inner hind tibial spur 0.2 times as long as basitarsus (3:14).

*Metasoma.* First-second with median carinae; first tergite 1.2 times as long as apical width (28:22); medial length of second tergite 1.7 times third (25:15; Fig. 10); second suture crenulate; third and fourth with basal striation, granulate apically; ovipositor sheath 2.5 times inner hind spur (15:6); ovipositor rather slender, gradually curved (Fig. 30).

Colour. Yellow; antenna yellow, medial and subapical segments with medial pale band; palpi pale yellow; stemmaticum black; side of scutellum and axilla brown; metasoma yellow except first tergite basally and sixth tergite medially brown; propodeum dark brown but yellow medially; wings subhyaline.

##### Male.

Unknown.

##### Variation.

Antennal segments 44(1); first tergite 1.1–1.2 times as long as apical width; medial length of metasomal second tergite 1.6–1.7 times as long as third tergite medially; body length 4.4–5.6 mm; fore wing length 3.4–4.3 mm.

##### Etymology.

From ‘eurys’, Greek for ‘widespread’.

#### Canalirogas hoabinhicus sp. n.

Taxon classificationAnimaliaHymenopteraBraconidae

http://zoobank.org/2AFF74EC-92C5-48B0-84A1-D12C17EB2C3F

Figs 6 , 32–37

##### Material.

Holotype, female (VNMN), ‘Rog.281’, “[NW Vietnam:] Hoa Binh, Yen Thuy, secondary forest, 20°23'N, 105°34'E, 315 m, MT, 20–30.viii.2002, KD Long”. Paratypes (3 females): 1 female (RMNH), ‘Rog.016’, id. but 5.v.2002; 1 female (VNMN), ‘Rog.692’, “[NW Vietnam:] Hoa Binh, Mai Chau, Tan Son, garden, 20°43'N, 105°59'E, 650 m, MT, 1–5.v.2010, KD Long”; 1 female (VNMN), ‘Rog.694’, id. but 20–25.viii.2010, KD Long.

##### Description.

Holotype, female, body length 6.4 mm, fore wing length 4.5 mm.

*Head.* Antenna incomplete, with 26 segments remaining; third segment 1.1 times fourth segment (9:8); middle segments 2.4 times longer than wide (6:2.5); width of face as long as length of face and clypeus combined; malar space 0.7 times as long as mandible width (4:6), mandible width 0.75 times as long as hypoclypeal depression (6:8); malar suture present; distance between tentorial pits 3.4 times distance between pits and eyes (17:5; Fig. 32); in dorsal view height of eye 3.3 times as long as temple (13:4); in lateral view width of eye about 3.0 times as long as temple (15:5); ocelli small, POL:Od:OOL = 4:5:3 (Fig. 34); distance between front and hind ocelli as long as OOL; face punctate; face shiny and sparsely punctate; frons, vertex and temple smooth.

*Mesosoma.* Length of mesosoma 1.32 times as long as high (33:25); pronotal trough mainly crenulate medially, finely granulate ventrally; precoxal sulcus narrow, rather long, crenulate; mesopleuron smooth dorsally, sparsely punctate ventrally (Fig. 33); metapleuron dull; mesoscutum dull because of irregular punctures; scutellar sulcus 0.6 times as long as scutellum (6:10); propodeum mainly rugose laterally and medial areola crenulate.

*Wings.* Fore wing: pterostigma 4.6 times as long as wide (41:9); r:2-SR:3-SR:SR1 = 8:14:23:37 (Fig. 36); vein r arising submedially from pterostigma; 1-CU1:cu-a:2-CU1:3-CU1 = 3:6:24:4; posterior length of second submarginal cell 2.8 times its apical width (37:13; Fig. 36). Hind wing: vein M+CU:1-M:1r-m = 31:26:10 (Fig. 37).

*Legs.* Hind coxa with sparse fine punctures; length of hind femur:tibia:basitarsus:tarsus = 57:74:35:87; length of hind femur, tibia and basitarsus 6.3, 9.3 and 11.7 times their width, respectively; inner hind tibial spur 0.3 times as long as basitarsus (10:35).

*Metasoma.* First tergite 1.3 times as long as apical width (35:27); medial length of second tergite 1.6 times third (31:19) (Fig. 6); second suture crenulate; third metasomal tergites obliquely striate, striation diverging posteriorly; fourth-sixth metasomal tergites obliquely striate basally, rugose-punctate apically; ovipositor sheath 0.7 times as long as hind basitarsus (23:35), ovipositor weakly curved and slender (Fig. 35).

*Colour.* Yellow; antenna and palpi yellow; stemmaticum dark brown; propodeum brown laterally; all legs yellow, but telotarsi brown; all metasomal tergites yellow, but first-second tergites basally and sixth tergite brown and hypopygium somewhat infuscate (Fig. 35); wings subhyaline.

##### Male.

Unknown.

##### Variation.

Antennal segments 52(1); first tergite 1.3–1.4 times as long as apical width; medial length of metasomal second tergite 1.5–1.7 times as long as third tergite medially; body length 6.0–7.5 mm; fore wing length 4.3–5.0 mm.

##### Etymology.

Named after its type locality: the province of Hoa Binh.

#### Canalirogas intermedius sp. n.

Taxon classificationAnimaliaHymenopteraBraconidae

http://zoobank.org/945FE282-E2D7-4127-99E3-40DCA68C2211

Figs 8 , 38–43

##### Material.

Holotype, female (VNMN) ‘Rog.589’, “[C Vietnam:] Thua Thien-Hue, Bach Ma NP, secondary forest, 300 m, 18.v.2007, KD Long”.

##### Description.

Holotype, female, body length 6.4 mm, fore wing length 4.8 mm, antenna 7.4 mm.

*Head.* Antenna with 51 segments, 1.2 times longer than body; third segment 1.3 times fourth segment (10:8); middle segments 3.5 times longer than wide (7:2), penultimate antennal segment as long as apical segment; apical segment with spine; width of face 0.9 times length of face and clypeus combined (16:18); malar space 0.5 times as long as mandible width (3:6), mandible width about 0.9 times as long as hypoclypeal depression (6:7); malar suture present; distance between tentorial pits 4.0 times distance between pits and eyes (8:2; Fig. 38); in dorsal view height of eye 4.5 times as long as temple (18:4); in lateral view width of eye 4.0 times as long as temple (16:4) ocelli large, POL:Od:OOL = 3:6:4 (Fig. 40); distance between front and hind ocelli 0.75 times OOL (3:4); face sparsely and finely punctate; frons, vertex and temple smooth.

*Mesosoma.* Length of mesosoma 1.5 times as long as high (66:45); pronotal side smooth dorsally and posteriorly, crenulate medio-anteriorly, finely granulate ventrally; precoxal sulcus narrow and sparsely crenulate; mesopleuron shiny and smooth; metapleuron smooth with sparse fine punctures (Fig. 39); notauli wide and crenulate anteriorly, flat and smooth posteriorly; scutellar sulcus 0.9 times scutellum (6:7); mesoscutum smooth; propodeum rugose laterally, with medial crenulate areola.

*Wings.* Fore wing: pterostigma 4.9 times as long as wide (44:9); r:2-SR:3-SR:SR1 = 9:12:25:40; vein r arising before middle of pterostigma (Fig. 42); vein 1-CU1 rather short, 1-CU1:cu-a:2-CU1:3-CU1 = 2:5.5:27:5; posterior length of second submarginal cell 3.1 times its apical width (Fig. 42). Hind wing: vein M+CU:1-M: 1r-m = 32:23:12 (Fig. 43).

*Legs.* Hind coxa with sparse fine punctures; length of hind femur:tibia:basitarsus:tarsus = 57:73:35:85; length of hind femur, tibia and basitarsus 6.3, 10.4 and 10.0 times as long as their width, respectively; inner hind tibial spur 0.3 times as long as basitarsus.

*Metasoma.* First tergite 1.4 times as long as apical width (30:19); medial length of second tergite 1.8 times as long as third (30:17; Fig. 8); third-fifth metasomal tergites with divergent striation; sixth tergite rugose-punctate; ovipositor sheath 0.5 times as long as hind basitarsus (19:35; Fig. 41).

*Colour.* Yellow; antenna and palpi yellow; stemmaticum black; propodeum blackish brown laterally, yellow medially and posteriorly; first metasomal tergite brown, but yellow apically; second-sixth metasomal tergites brown, but lateral corners yellow; fore wing yellow with veins 1-M, 2-CU1 and CU1a medially, veins r and 2-SR brown; pterostigma brown medially, yellow basally and apically; pronotum, mesopleuron and metapleuron ivory; middle and lateral lobes of mesoscutum and side of scutellum yellow; outer side of hind coxa and hind femur subapically dark yellow.

##### Male.

Unknown.

##### Etymology.

From ‘inter’ (Latin for ‘between’), because this species is intermediate between *Canalirogas parallelus* sp. n. and *Canalirogas spilonotus* (Cameron), but differs from these species by having larger occelli (diameter of posterior ocellus 3.0 times as long as POL and 1.5 times as long as OOL). This species is close to *Canalirogas spilonotus*, but differs by having the mesopleuron antero-dorsally and below the precoxal sulcus pale yellow (dark brown antero-dorsally and more or less brownish below precoxal sulcus in *Canalirogas spilonotus*), the ovipositor sheath entirely brown (only apically dark brown) and the third and fourth metasomal tergites with nearly transverse striation apically (absent).

#### Canalirogas parallelus sp. n.

Taxon classificationAnimaliaHymenopteraBraconidae

http://zoobank.org/FCCA8CAC-7D22-480E-B361-47C54D49AE88

Figs 4 , 5 , 44–48

##### Material.

Holotype, female (RMNH), “[S Vietnam:] Kon Tum, Chu Mom Ray NP, Mal. traps, 700–900 m, 26.ix–5.x.2006, Mai Phu Quy & Nguyen Thanh Manh, RMNH’07”. Paratype, 1 female (VNMN), ‘Rog.520’, “[NE Vietnam:] Ha Giang, Vi Xuyen, Cao Bo, forest, 300 m, 11.v.2007, KD Long”.

Excluded from type series a female from Central Vietnam (missing its metasoma; IEBR) ‘Rog.590’, ‘C. Vietnam: Thua Thien-Hue, Bach Ma NP, secondary forest 300 m, 20.v.2007, KD Long’ with the precoxal area dark brown.

##### Description.

Holotype, female, body length 7.2 mm, fore wing length 5.2 mm, antenna 10.2 mm.

*Head.* Antenna with 57 segments, 1.4 times longer than body; third segment 1.1 fourth segment (9:8); middle segment 2.7 times as long as wide (8:3), penultimate antennal segment 0.75 times apical segment (6:8); apical segment with spine; width of face 0.9 times length of face and clypeus combined (28:21); malar space 0.7 times as long as mandible width (4:6); basal width of mandible 0.7 times as long as hypoclypeal depression (6:9); malar suture present; distance between tentorial pits 3.0 times distance between pits and eyes (9:3; Fig. 44); in dorsal view height of eye 5.0 times as high as temple (20:4); in lateral view width of eye 3.4 times as long as temple (17:5); ocelli in high triangle, POL:Od:OOL = 4:6:4 (Fig. 46); distance between front and hind ocelli as long as OOL; face rugose-punctate; frons, vertex and temple smooth.

*Mesosoma.* Length of mesosoma 1.45 times as long as high (77:54); pronotal side mainly crenulate medially smooth dorsally, finely granulate ventrally; notauli deep and long, punctate; scutellar sulcus 0.55 times as long as scutellum; mesopleuron and metapleuron smooth; precoxal sulcus rather wide and crenulate (Fig. 45); propodeum mainly rugose laterally and medial areola crenulate (Fig. 5).

*Wings.* Fore wing: pterostigma 4.8 times as long as wide; r:2-SR:3-SR:SR1 = 9:14:27:43; vein r arising before middle of pterostigma; 1-CU1:cu-a:2-CU1:3-CU1=4:7:27:5; posterior length of second submarginal cell 2.5 times its apical width (33:13). Hind wing: vein M+CU:1-M:1r-m = 35:24:11 (Fig. 48).

*Legs.* Hind coxa almost smooth; length of hind femur:tibia:basitarsus:tarsus = 62:85:44:108; length of hind femur, tibia and basitarsus 6.2, 10.6 and 11.0 times as long as their width, respectively; inner hind tibial spur 0.25 times as long as basitarsus (11:44).

*Metasoma.* First tergite 1.7 times as long as apical width (45:27; Fig. 4); medial length of second tergite 1.6 times as long as third (34:21); second suture crenulate; second metasomal tergite obliquely and longitudinally striate; basal area of third-fifth metasomal tergites with divergent striation, apex of third-fifth metasomal tergites with curved striation mixing with punctures (Fig. 4); ovipositor sheath 0.5 times as long as hind basitarsus (1:2; Fig. 47).

*Colour.* Pale yellow; antenna yellowish brown, basal antennal segments with medial pale band; palpi yellow; stemmaticum black; propleuron, mesopleuron anteriorly, side of scutellum and axilla, metanotum and propodeum entirely black; second-sixth metasomal segments black, yellow laterally; hypopygium yellow, brownish ventrally; all legs yellow, but hind coxa yellowish brown ventrally; wings subhyaline with veins brownish yellow; parastigma yellow; pterostigma mainly brown, yellow subapically.

##### Male.

Unknown.

##### Etymology.

Named ‘parallelus’, because of the nearly parallel-sided first metasomal tergite.

#### Canalirogas robberti sp. n.

Taxon classificationAnimaliaHymenopteraBraconidae

http://zoobank.org/CC50053B-5145-4724-B6CE-670BAD92E6FF

Figs 49–56

##### Material.

Holotype, female (RMNH), “[S Vietnam:] Dông Nai, Cát Tiên NP, *Ficus* trail, Mal. trap[s] 1-8, c. 100 m, 1-9.x.2005, C. v. Achterberg & R. de Vries, RMNH’05”.

##### Description.

Holotype, female, body length 7.7 mm, fore wing length 5.4 mm.

*Head.* Antenna with 52 segments, 1.8 times as long as fore wing; middle and subapical segments 2.6 and 2.5 times longer than wide, respectively; third antennal segment 1.1 times as long as fourth segment; width of face 0.8 times length of face and clypeus combined; clypeus concave medially in lateral view, with distinct ventral rim (Fig. 51); malar space 0.6 times as long as basal width of mandible; basal width of mandible 0.7 times as long as width of hypoclypeal depression; malar suture deep; distance between tentorial pits 3.4 times distance between pits and eyes (Fig. 50); length of eye in dorsal view 5.5 times as long as temple (Fig. 53); width of eye in lateral view 3.8 times as long as temple; ocelli large, POL:Od:OOL = 1:3:1; distance between front and hind ocelli as long as OOL (Fig. 53); face with some distinct punctures laterally, with some indistinct rugae sublaterally, remainder of face, frons, vertex and temple smooth.

*Mesosoma.* Length of mesosoma 1.3 times as long as high; pronotal side smooth dorsally, coarsely crenulate medially and superficially granulate and with some rugae ventrally; precoxal sulcus only posteriorly absent and finely crenulate (Fig. 51); mesopleuron and metapleuron mainly smooth; mesoscutum smooth, except some punctulation; notauli narrow, shallow posteriorly and finely crenulate; scutellar sulcus 0.5 times as long as scutellum and with one long crenula (Fig. 52); scutellum smooth except some striae posteriorly; propodeum rugulose-granulate dorsally, except carinate median areola and some coarse rugae posteriorly (Fig. 52).

*Wings.* Fore wing: pterostigma 4.8 times as long as wide; r:2-SR:3-SR:SR1 = 5:8:14:22; vein r emerging before middle of pterostigma; vein cu-a slender (Fig. 49), 1-CU1:cu-a:2-CU1:3-CU1 = 3:10:38:8; posterior length of second submarginal cell 3.1 times its apical width. Hind wing: vein M+CU:1-M: 1r-m = 15:12:6; vein SR unsclerotised.

*Legs.* Hind coxa shiny and with sparse fine punctures; length of hind femur:tibia:basitarsus: tarsus = 60:81:38:94; length of hind femur, tibia and basitarsus 6.0, 10.0 and 9.6 times as long as their width, respectively (Fig. 55); inner hind tibial spur 0.3 times as long as basitarsus.

*Metasoma.* First tergite 1.7 times as long as apical width and slightly widened posteriorly (Fig. 54); first-second tergites with costate and somewhat oblique striation; third-fifth tergites with finer and more divergent striation and sixth tergite finely rugulose; medial length of second tergite 1.7 times than third segment; second suture coarsely crenulate; ovipositor sheath truncate apically and half as long as hind basitarsus; ovipositor stout (Fig. 56).

*Colour.* Pale yellow or ivory; antennal segments pale brown with faint yellowish transverse bands; stemmaticum, propleuron partly, mesopleuron antero-dorsally, antero-ventrally and below precoxal sulcus, mesoscutum laterally, scutellum and metanotum laterally, propodeum (except areola and posteriorly), inner and outer side of hind coxa, outer and inner side of hind femur mainly (except basally), metasomal tergites 1-5 basally and medio-posteriorly, tergite 6 nearly entirely dorsally (Fig. 54) and ovipositor sheath (except basally), dark brown; telotarsi and hypopygium baso-ventrally brown; wings mainly slightly infuscate; veins mainly (but of apical third of wing brownish yellow) and pterostigma medially dark brown; remainder of pterostigma and parastigma yellow.

##### Male.

Unknown.

##### Etymylogy.

Named after one of the collectors of the holotype, Mr. Rob de Vries (Leiden); for his excellent collaboration.

#### Canalirogas spilonotus

Taxon classificationAnimaliaHymenopteraBraconidae

(Cameron, 1905)

Figs 57–64

Troporhogas spilonotus Cameron, 1905: 93. Lectotype female (BMNH: Hym. Type 3c.222 from Sri Lanka) examined and here designated.
Canalirogas spilonotus Quicke & Shaw, 2005: 3531.
Canalirogas balgooyi van Achterberg & Chen, 1996: 70–73 (description). **Syn. n.**

##### Material.

Specimens examined from North and North Central and South Vietnam (IEBR, RMNH and VNMN): Ha Giang (Vi Xuyen), Hoa Binh (Mai Chau, Yen Thuy), Ninh Binh (Cuc Phuong NP), Ha Tinh (Huong Son, Vu Quang NP), Phu Tho (Tan Son), Vinh Phuc (Me Linh; Tam Dao NP), Dông Nai (Cat Tien NP).

##### Description.

Figured female from Cát Tiên National Park, body length 7.1 mm, fore wing length 5.5 mm.

*Head.* Antenna with 51 segments, 1.7 times as long as fore wing; middle and subapical segments 2.6 and 2.5 times longer than wide, respectively; third antennal segment 1.3 times as long as fourth segment; width of face 0.8 times length of face and clypeus combined; clypeus flat in lateral view (Fig. 59); malar space 0.6 times as long as basal width of mandible; basal width of mandible 0.7 times as long as width of hypoclypeal depression; malar suture deep; distance between tentorial pits 3.9 times distance between pits and eyes (Fig. 58); length of eye in dorsal view 7.8 times as long as temple (Fig. 61); width of eye in lateral view 5.6 times as long as temple; ocelli large, POL:Od:OOL = 5:14:5; distance between front and hind ocelli as long as OOL (Fig. 61); face weakly rugose sublaterally, remainder of face, frons, vertex and temple smooth.

*Mesosoma.* Length of mesosoma 1.3 times as long as high; pronotal side smooth dorsally, coarsely crenulate medially and superficially granulate ventrally; precoxal sulcus only posteriorly absent and finely crenulate (Fig. 59); mesopleuron and metapleuron largely smooth; mesoscutum smooth, except some punctulation; notauli narrow, shallow posteriorly and finely crenulate; scutellar sulcus 0.6 times as long as scutellum and with 3 long crenulae (Fig. 60); scutellum smooth except some striae posteriorly; propodeum densely finely punctate dorsally, except carinate median areola and laterally rugose (Figs 60, 62).

*Wings.* Fore wing: pterostigma 4.9 times as long as wide; r:2-SR:3-SR:SR1 = 10:14:18:43; vein r emerging little before middle of pterostigma; vein cu-a short and slender (Fig. 57), 1-CU1:cu-a:2-CU1:3-CU1 = 1:5:24:4; posterior length of second submarginal cell 3.4 times its apical width. Hind wing: vein M+CU:1-M: 1r-m = 30:26:16; vein SR unsclerotised.

*Legs.* Hind coxa shiny and with sparse fine punctures; length of hind femur:tibia:basitarsus: tarsus = 50:64:31:78; length of hind femur, tibia and basitarsus 6.2, 11.4 and 11.6 times as long as their width, respectively (Fig. 63); inner hind tibial spur 0.3 times as long as basitarsus.

*Metasoma.* First tergite 1.5 times as long as apical width and slightly widened posteriorly (Fig. 62); first-third tergites with costate and somewhat oblique striation; fourth-fifth tergites with more divergent striation; medial length of second tergite 1.6 times than third segment; second suture crenulate; ovipositor sheath truncate apically and half as long as hind basitarsus; ovipositor rather stout (Fig. 64).

*Colour.* Pale yellow or ivory; antennal segments pale brown with yellow transverse bands (Fig. 58); stemmaticum, propleuron partly, mesopleuron antero-dorsally, mesoscutum laterally, metanotum partly laterally, propodeum (except areola, narrowly posteriorly and partly latero-posteriorly), inner and outer side of hind coxa, metasomal tergites 1-5 basally and medio-posteriorly (but of third-fifth tergites partly brown antero-laterally), tergite 6 entirely dorsally (Fig. 57) and apex of ovipositor sheath narrowly dark brown (Fig. 64); telotarsi brown; wings largely slightly infuscate; veins (but of apical third of wing unpigmented) and pterostigma medially dark brown; remainder of pterostigma and parastigma yellow.

##### Male.

Unknown.

##### Variation.

Antennal segments of female 44(1), 48(1), 50(1) or 51(1); first tergite 1.2–1.5 times as long as apical width (Fig. 57); medial length of second tergite 1.5–1.6 times as long as third tergite medially; body length 6.2–7.1 mm; fore wing length 4.6–5.5 mm.

##### Notes.

This conspicuous species has the eyes in dorsal view 6–8 times longer than temple (Fig. 61) and in lateral view width of eye about 3.8 times temple (15:4; Fig. 59); ovipositor stout (Fig. 64). The lectotype of *Canalirogas spilonotus* (Cameron) designated in this paper falls within the (rather wide) variation limits of *Canalirogas balgooyi* and is, therefore, considered to be a senior synonym of the latter.

#### Canalirogas vittatus sp. n.

Taxon classificationAnimaliaHymenopteraBraconidae

http://zoobank.org/3D065D35-E536-4EBB-AE82-48F81FD46775

Figs 7 , 65–70

##### Material.

Holotype, female (VNMN) ‘Rog.014’, “[NE Vietnam:] Ninh Binh, Cuc Phuong NP, 7–9.v.2002, KD Long”. Paratypes, 2 females (RMNH, VNMN), ‘Rog.013’ & ‘Rog.005’, topotypic and same date.

##### Description.

Holotype, female, body length 5.9 mm, fore wing length 5.0 mm, antenna 8.0 mm.

*Head.* Antenna with 47 segments, 1.4 times longer than body; third segment 1.1 times fourth; middle segments 2.8 times longer than wide (7:2.5); penultimate antennal segment 0.9 times apical segment; apical segment with spine; width of face slightly less than length of face and clypeus combined (18:19); malar space 0.7 times as long as mandible width (5:7); basal width of mandible 0.8 times as long as hypoclypeal depression (7:9); malar suture absent; distance between tentorial pits 3.0 times distance between pits and eyes (9:3; Fig. 68); in dorsal view height of eye 3.2 times as high as temple (16:5); in lateral view width of eye 2.5 times as long as temple (10:4; Fig. 65); ocelli rather small and in high triangle, POL:Od:OOL = 4:5.5 (Fig. 67); distance between front and hind ocelli 0.8 times OOL (4:5); face rugose-punctate; frons, vertex and temple smooth.

*Mesosoma.* Length of mesosoma 1.35 times longer than high (69:51); pronotal side mainly crenulate medially, granulate ventrally; precoxal sulcus wide and punctate-crenulate (Fig. 66); mesopleuron smooth dorsally and punctate ventrally, punctures merged into mesosternum; scutellum smooth; mesopleuron smooth, rugose anteriorly; notauli deep, crenulate; mesoscutum with sparse fine punctures; scutellar sulcus 0.7 times as long as scutellum (6:9); propodeum mainly rugose laterally and its medial areola crenulate.

*Wings.* Fore wing: pterostigma 4.7 times as long as wide (42:9); r:2-SR:3-SR:SR1 = 9:15:29:44; vein r arising before middle of pterostigma; 1-CU1:cu-a:2-CU1:3-CU1 = 4:7:27:5; ventral length of second submarginal cell 3.4 times its apical width (41:12). Hind wing: vein M+CU:1-M:1r-m = 31:26:23 (Fig. 70).

*Legs.* Hind coxa shiny with sparse fine punctures; length of hind femur:tibia:basitarsus:tarsus = 55:74:38:89; length of hind femur, tibia and basitarsus 5.5, 9.25 and 9.5 times as long as their width, respectively; inner hind tibial spur 0.24 times as long as basitarsus (9:38).

*Metasoma.* First tergite subequal to apical width; medial length of second tergite 1.6 times third (30:19; Fig. 7); second suture crenulate; second tergite with parallel striation; third-fifth tergites mainly rugose medially; sixth tergite with curved fine striation mixed with granulation; ovipositor sheath 0.5 times as long as hind basitarsus (18:38; Fig. 69).

*Colour.* Yellow; antennal segments brown with medial pale band; palpi pale yellow; stemmaticum black; occipital carina brown; propleuron, mesopleuron anteriorly, precoxal sulcus, notauli, mesonotum laterally, side of scutellum and axilla black; propodeum black, but pale yelllow medially; fore and middle legs yellow, except middle femur subapically and tarsus darker than tibia; hind coxa blackish brown, except yellow dorso-basally; hind trochantellus, most part of hind femur and hind tarsus brown; hind tibia dirty yellow; wings dirty subhyaline with pterostigma and veins brown, except veins 3-SR, SR1, 3-M and r-m yellow; first-fifth metasomal tergites black, yellow laterally and at posterior corners; sixth tergite entirely black.

##### Male.

Unknown.

##### Variation.

Length of first metasomal tergite 1.0–1.2 times as long as apical width; medial length of metasomal second tergite 1.6–1.7 times as long as third tergite medially; body length 5.1–6.2 mm; fore wing length 4.0–5.1 mm.

##### Etymology.

From ‘vitta’ (Latin for ‘ribbon, band’), because of the pale band of the antennal segments.

#### Canalirogas vuquangensis sp. n.

Taxon classificationAnimaliaHymenopteraBraconidae

http://zoobank.org/99337E9B-C176-4967-A419-6A0352BF4BA4

Figs 1 , 71–78

##### Material.

Holotype, female (RMNH), “[C Vietnam:] Ha Tinh, Vu Quang NP, 66 m, 18°19'47"N, 105°26'28"E, Mal. trap 9, 4.iii–15.iv.2011, C. v. Achterberg, RMNH’11”.

##### Description.

Holotype, female, body length 6.6 mm, fore wing length 4.8 mm.

*Head.* Antenna with 44 segments, 1.6 times as long as fore wing; middle and subapical segments 3.3 and 2.7 times longer than wide, respectively; third antennal segment 1.4 times as long as fourth segment; width of face 0.8 times length of face and clypeus combined; clypeus flat in lateral view and ventral rim not differentiated from clypeus (Fig. 73); malar space 0.7 times as long as basal width of mandible; basal width of mandible 0.7 times as long as width of hypoclypeal depression; malar suture deep; distance between tentorial pits 2.9 times distance between pits and eyes (Fig. 72); length of eye in dorsal view 8.3 times as long as temple (Fig. 75); width of eye in lateral view 4.4 times as long as temple; ocelli large, POL:Od:OOL = 2:6:3; distance between front and hind ocelli as long as OOL (Fig. 72); face distinctly granulate submedially and orbita sparsely punctate, remainder of face superficially coriaceous; frons, vertex and temple smooth.

*Mesosoma.* Length of mesosoma 1.4 times as long as high; pronotal side smooth dorsally, moderately crenulate medially and granulate ventrally; precoxal sulcus only medially distinctly impressed and finely crenulate; mesopleuron mainly smooth; metapleuron superficially granulate (Fig. 73); mesoscutum smooth, except some punctulation; notauli narrow, shallow posteriorly and smooth; scutellar sulcus 0.5 times as long as scutellum and with one long crenula; scutellum smooth except some striae posteriorly; propodeum distinctly granulate dorsally, except carinate median areola, rugose medially and superficially granulate posteriorly (Figs 73, 74).

*Wings.* Fore wing: pterostigma 4.9 times as long as wide; r:2-SR:3-SR:SR1 = 5:9:15:26; vein r emerging before middle of pterostigma; vein cu-a slender (Fig. 71), 1-CU1:cu-a:2-CU1:3-CU1 = 3:8:34:7; posterior length of second submarginal cell 3.1 times its apical width. Hind wing: vein M+CU:1-M: 1r-m = 15:13:7; vein SR unsclerotised.

*Legs.* Hind coxa with satin sheen, superficially coriaceous and punctulate; length of hind femur:tibia:basitarsus:tarsus = 50:74:33:78; length of hind femur, tibia and basitarsus 6.0, 10.3 and 11.6 times as long as their width, respectively (Fig. 77); inner hind tibial spur 0.3 times as long as basitarsus.

*Metasoma.* First tergite 1.9 times as long as apical width and slightly widened posteriorly (Fig. 76); first-second tergites with rather coarse and somewhat oblique rugae; third-fifth tergites with more divergent rugulae and sixth tergite mainly coriaceous; medial length of second tergite 1.7 times longer than of third segment; second suture moderately crenulate; ovipositor sheath truncate apically and 0.6 times as long as hind basitarsus; ovipositor moderately stout (Fig. 78).

*Colour.* Pale yellow or ivory; antennal segments brown with yellowish transverse bands (Fig. 1); stemmaticum and face sublaterally pale brown; scapus, pedicellus, telotarsi, inner side of hind coxa, patch on outer side and inner side of hind femur, ovipositor sheath (except basally) and hypopygium baso-ventrally dark brown; propleuron partly, mesopleuron antero-dorsally, antero-ventrally and below precoxal sulcus, mesoscutum laterally, notaulic courses, scutellum and metanotum laterally, propodeum (except areola and posteriorly), outer side of hind coxa, metasomal tergites 1–3 basally and medio-posteriorly and tergites 4–6 nearly entirely dorsally (Figs 1, 76) blackish brown; wings largely slightly infuscate; veins mainly (but of apical third of wing brownish yellow) and pterostigma medially and subbasally dark brown; remainder of pterostigma and parastigma yellow.

##### Male.

Unknown.

##### Etymylogy.

Named after the type locality in Central Vietnam.

## Supplementary Material

XML Treatment for Canalirogas

XML Treatment for Canalirogas affinis

XML Treatment for Canalirogas cucphuongensis

XML Treatment for Canalirogas curvinervis

XML Treatment for Canalirogas eurycerus

XML Treatment for Canalirogas hoabinhicus

XML Treatment for Canalirogas intermedius

XML Treatment for Canalirogas parallelus

XML Treatment for Canalirogas robberti

XML Treatment for Canalirogas spilonotus

XML Treatment for Canalirogas vittatus

XML Treatment for Canalirogas vuquangensis

## References

[B1] Achterberg C van (1988) Revision of the subfamily Blacinae Foerster (Hymenoptera, Braconidae). Zoologische Verhandelingen Leiden 249: 1–324.

[B2] Achterberg C van (1990) Illustrated key to the subfamilies of the Holarctic Braconidae (Hymenoptera: Ichneumonoidea). Zoologische Mededelingen Leiden 64: 1–20.

[B3] Achterberg C van (1991) Revision of the genera of the Afrotropical and W. Palaearctic Rogadinae Foerster (Hymenoptera: Braconidae). Zoologische Verhandelingen Leiden 273: 1–102.

[B4] Achterberg C van (1993) Illustrated key to the subfamilies of the Braconidae (Hymenoptera: Ichneumonoidea). Zoologische Verhandelingen Leiden 283: 1–189.

[B5] Achterberg C van Chen X (1996) *Canalirogas*, a new genus of the subfamily Rogadinae Foerster (Hymenoptera: Braconidae) from the Indo-Australian region. Zoologische Mededelingen Leiden 70(3): 63–92.

[B6] Achterberg C van (1997) Braconidae. An illustrated key to all subfamilies. ETI World Biodiversity Database CD-ROM Series Amsterdam.

[B7] Cameron P (1905) On the phytophagous and parasitic Hymenoptera collected by Mr. E.Green in Ceylon. Spolia Zeylanica 3: 67–143.

[B8] Chen X-X He J-H (1997) Revision of the subfamily Rogadinae (Hymenoptera: Braconidae) from China. Zoologische Verhandelingen Leiden 308: 1–187.

[B9] Quicke DLJ Shaw MR (2005) First host records for the rogadine genera *Rogasodes* Chen and He and *Canalirogas* van Achterberg and Chen (Hymenoptera: Braconidae) with description of a new species and survey of mummy types within Rogadinae s. str. Journal of Natural History 39(40): 3525–3542. doi: 10.1080/00222930500392782

[B10] Yu DS Achterberg K van Horstmann K (2012) Biological and taxonomical information: Ichneumonoidea 2012. Taxapad Interactive Catalogue, Ottawa.

